# Divalent Metal Ion Coordination by Residue T118 of Anthrax Toxin Receptor 2 Is Not Essential for Protective Antigen Binding

**DOI:** 10.1371/journal.pone.0000099

**Published:** 2006-12-20

**Authors:** Heather M. Scobie, John A.T. Young

**Affiliations:** Infectious Disease Laboratory, The Salk Institute for Biological Studies, La Jolla, California, United States of America; The Research Institute for Children, United States of America

## Abstract

The protective antigen (PA) subunit of anthrax toxin interacts with the integrin-like I domains of either of two cellular receptors, ANTXR1 or ANTXR2. These I domains contain a metal ion-dependent adhesion site (MIDAS) made up of five non-consecutive amino acid residues that coordinate a divalent metal ion that is important for PA-binding. The MIDAS residues of integrin I domains shift depending upon whether the domain exists in a closed (ligand-unbound) or open (ligand-bound) conformation. Of relevance to this study, the MIDAS threonine residue coordinates the metal ion only in the open I domain conformation. Previously it was shown that the MIDAS threonine is essential for PA interaction with ANTXR1, a result consistent with the requirement that the I domain of that receptor adopts an open conformation for PA-binding [1]. Here we have tested the requirement for the MIDAS threonine of ANTXR2 for PA-binding. We show that the toxin can bind to a mutant receptor lacking the MIDAS threonine and that it can use that mutant receptor to intoxicate cultured cells. These findings suggest that an open-like configuration of the ANTXR2 MIDAS is not essential for the interaction with PA.

## Introduction

Anthrax toxin is an AB-type toxin, secreted by *B. anthracis* that contributes to bacterial virulence, as well as causing many disease symptoms. The toxin has a single B-moiety, protective antigen (PA), for receptor binding and cytoplasmic delivery of the two catalytic A-moieties, lethal factor (LF) and edema factor (EF) [2]. The first step of intoxication involves binding of the full-length, 83 kDa form of PA (PA_83_) to either of two cell surface receptors, ANTXR1 (anthrax toxin receptor/tumor endothelial marker 8; ATR/TEM8) or ANTXR2 (capillary morphogenesis gene 2; CMG2) [3], [4]. These receptors share a von Willebrand factor A (VWA) or integrin inserted (I) domain, containing a metal ion dependent adhesion site (MIDAS), which binds PA. The MIDAS is made up of five non-consecutive, metal ion-coordinating, residues, i.e. D50, S52, S54, T118, and D148 in the case of ANTXR2.

Ligand binding to α-integrin I domains is regulated by a structural change from a “closed” or ligand-unbound conformation to an “open” or ligand-bound conformation [5]. These two conformations are associated with changes in divalent metal cation coordinating residues. In the closed conformation, ion coordination is mediated by both MIDAS serines, by the second MIDAS aspartate, and by three water molecules. In the open conformation, the ion is instead coordinated by a carboxylate-containing ligand side-chain, both MIDAS serines, the MIDAS threonine, and two water molecules. By coordinating the metal ion only in the open conformation, the MIDAS threonine residue is thought to increase the electrophilicity of the metal and therefore affinity for ligand, making it critical for the structural transition between the closed and open states of the I domain [6]. The other major structural change that accompanies this transition is a 10Å translocation of the I domain C-terminal α-helix [5].

**Figure 1:**
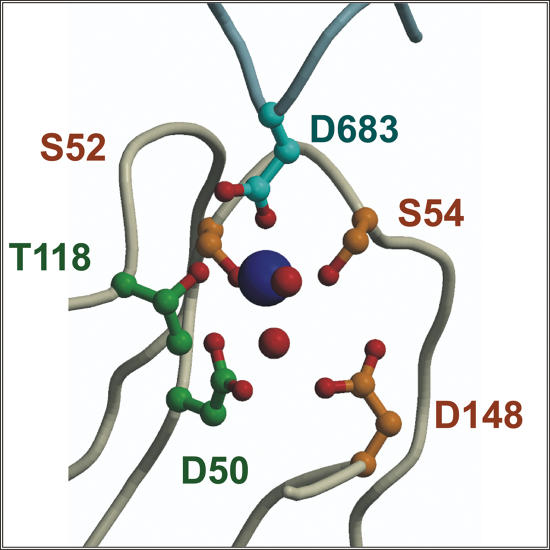
ANTXR2 MIDAS metal ion bound by PA D683. Close-up view of the PA-ANTXR2 generated with Molscript (PDB # ITZN) [7]. The PA backbone is colored light blue with the D683 residue shown in cyan. The chelate Mg^2+^ ion is colored purple, the ANTXR2 Thr118 and Asp50 residues (mutated in this report) are shown in green with the other MIDAS residues represented in orange. Water molecules and oxygen atoms are represented by large and small red balls, respectively.

The crystal structure of the ANTXR2-PA complex has revealed that PA–receptor binding appears to resemble ligand binding to the open conformation of α-integrin I domains. Namely, the ANTXR2-bound divalent cation is directly coordinated by a carboxylate-containing side chain (residue D683) of PA as well as by the MIDAS threonine and serine residues, and water molecules coordinated by the MIDAS aspartate residues (Fig. 1) [7], [8]. However, the ANTXR2-PA binding surface which includes residues from the receptor's I domain MIDAS face and residues from PA domains 2 and 4 (∼2000Å^2^) is much larger than that of α-integrin-ligand interactions (∼1300 Å^2^) [7], [8].

Previously it was shown that, similar to α-integrin-ligand interactions, the first MIDAS aspartate (D50) and the MIDAS threonine (T118) are critically important for PA binding and intoxication via ANTXR1 [1]. Mutation of the first MIDAS aspartate abolishes metal ion binding whereas mutation of the MIDAS threonine, while permitting metal ion binding, prevents the MIDAS from adopting the configuration associated with the open I domain conformation [6]. Given the importance of residue T118 of ANTXR1 it was proposed that an open-like configuration of the I domain of that receptor is essential for PA-binding [1].

In this report, we have tested whether the same holds true for ANTXR2 and we show that, unlike in the case of ANTXR1, residue T118 is not essential for the ANTXR2-PA interaction, indicating that the I domain of ANTXR2 does not need to adopt a classical open-like conformation to bind PA.

## Materials and Methods

### DNA constructs and cell lines

Quikchange mutagenesis (Stratagene) performed on the previously described ANTXR2-enhanced green fluorescent protein (EGFP) fusion construct (CMG2^489^-EGFP) and soluble ANTXR2-mycHis fusion constructs (sCMG2) [4], [9] to generate D50A and T118A mutations with the oligonucleotide primers 5′ CTT CGT CCT GGC AAA GTC TGG GAG TG 3′ and 5′ CCA GTA GGA GAG GCA TAT ATC CAT G 3′, respectively. The open reading frames of all constructs were confirmed by DNA sequencing.

PA receptor-deficient CHO-R1.1 cells, and CHO-R1.1 cells that were engineered to stably express CMG2^489^-EGFP have been described previously [4]. CHO-R1.1 cells transduced with retroviral vectors encoding the D50A or T118A mutant ANTXR2 proteins were either selected in medium containing G418 (Invitrogen) and/or enriched by flow cytometric sorting on the basis of EGFP expression [4].

### PA and sANTXR2 protein production

The WT and mutant sANTXR2-mycHis proteins were purified from the extracellular supernatants of transfected human 293Freestyle cells (Invitrogen) as described elsewhere [9]. PA was prepared from the periplasm of *E. coli* BL21 cells that had been transformed with the PA-Pet22b as described previously [9]. The PA protein was purified by FPLC with HiTrap QFF and Superose 12 (Amersham) or HiLoad Superdex 200 (Amersham) columns, and the relative protein purity was determined by ImageQuant analysis (Amersham) of coomassie-stained protein samples following SDS-PAGE.

### Cell surface receptor expression analysis

Cell surface expression of the mutant D50A and T118A ANTXR2 receptors was confirmed using a membrane-impermeable biotinylating reagent (EZ-Link SuLFo-NHS-Biotin; Pierce) and pelleting biotinylated proteins with immobilized streptavidin gel (Immunopure; Pierce) essentially as described elsewhere [1]. The pelleted samples were boiled in reducing SDS sample buffer and subjected to SDS-PAGE using a 10% polyacrylamide gel. The samples were then transferred to a PVDF membrane and immunoblotted with a 1∶5000 dilution of anti-GFP antibody (118P; Covance), followed by a 1∶2500 dilution of anti-mouse HRP (Dako), in TBST (100 mM Tris pH 7.5, 150 mM NaCl, 0.1% Tween-20) with 3% non-fat milk. For control purposes, CHO-R1.1 cells transduced with a retroviral vector encoding cytoplasmic EGFP from pLEGFP.C1 (Clontech) were also used to confirm that the biotinyation method used did not label cytoplasmic proteins (data not shown).

### PA binding and in vitro intoxication assays

PA binding to cells was monitored by flow cytometric analysis following incubations of cells for 2 hours with 100 nM PA, then with a 1∶2000 dilution of an anti-PA rabbit polyclonal serum, and a 1∶500 dilution of an APC-conjugated anti-rabbit antibody (Molecular Probes) [4]. Intoxication was monitored by incubating samples of cells with LF_N_-DTA [10] and PA. These experiments were performed either with, or without sANTXR2 inhibitor proteins. Cell viability was measured 25–30 hours later using the WST-1 reagent (Roche), or 45–50 hours later using the CellTiter-glo reagent (Promega) [4], [9]. The IC_50_ was determined as the concentration of inhibitor at the midpoint of the soluble receptor titration curve. In all intoxications, % cell viability is the average from three samples, plus or minus the S.D.

## Results and Discussion

### The T118A mutant receptor binds PA

**Figure 2:**
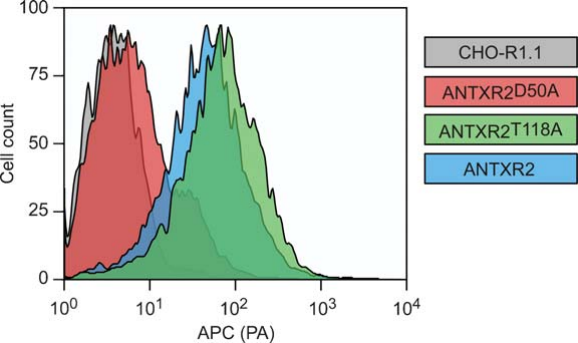
The cell surface T118A mutant ANTXR2 receptor binds PA. (A) Flow cytometric analysis of PA binding to CHO-R1.1 cells and CHO-R1.1 cells engineered to express WT or mutant ANTXR2-EGFP fusion proteins. The cells were incubated with PA and then with a PA-specific antibody followed by an APC-labeled secondary antibody.

To test the importance of ANTXR2 residue T118, it was replaced with an alanine in the context of a human ANTXR2-EGFP fusion protein [4]. For control purposes, residue D50 of ANTXR2 was also changed to an alanine (a mutation that leads to loss of metal ion binding) in ANTXR2-EGFP. The mutant ANTXR2^D50A^-EGFP and ANTXR2^T118A^-EGFP proteins were then expressed in the PA receptor-deficient cell line, CHO-R1.1 [3]. Flow cytometric analysis performed with PA, an anti-PA antibody and a fluoresceinated secondary antibody revealed that the T118A mutant receptor was expressed at the cell surface and could bind PA (Fig. 2). By contrast, the D50A mutant receptor did not bind PA (Fig. 2). Cell surface expression of the D50A and T118A mutant receptors was confirmed using a membrane-impermeable biotinylating agent as described elsewhere [1] (data not shown).

**Figure 3:**
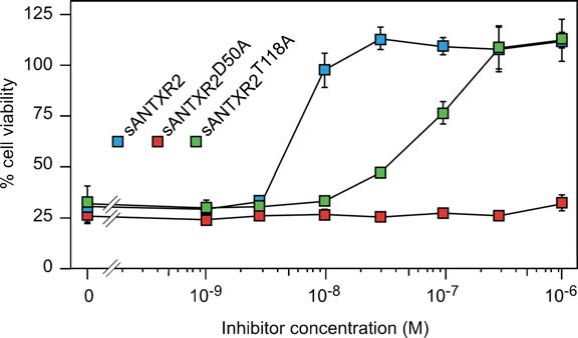
Soluble ANTXR2^T118A^ inhibits intoxication of cells. WT and mutant sANTXR2 proteins were added in increasing amounts to measure their relative abilities to block CHO-K1 cell intoxication with PA (10^−8^ M) and LF_N_-DTA (10^−10^ M). Cell viability was subsequently measured using the WST-1 assay and is represented as the percentage of that seen with cells incubated with LF_N_-DTA alone (100% viable).

As an independent measure of how the D50A and T118A mutations affect PA binding, these mutations were introduced independently into the soluble human ANTXR2 receptor-decoy protein (sANTXR2) which, through binding extracellular PA, can protect cells and animals against intoxication [4], [11]. The altered forms of sANTXR2 were tested for their abilities to protect CHO-K1 cells from intoxication by PA and LF_N_-DTA. Consistent with activity of the full-length T118A mutant and wild-type receptors, the sANTXR2^T118A^ inhibitor was ∼13-fold less efficient at blocking intoxication (IC_50_ = 80 nM) as compared to WT sANTXR2 (IC_50_ = 6 nM) (Fig. 3). As expected sANTXR2^D50A^ was not a functional receptor-decoy (Fig. 3). These results confirm that the T118A mutation receptor is still capable of PA binding.

### The T118A mutant receptor supports intoxication

**Figure 4:**
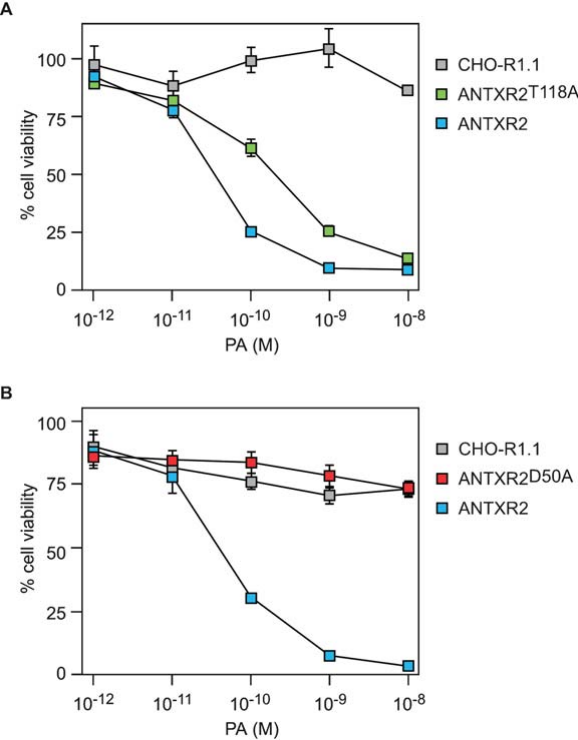
The cell surface T118A mutant ANTXR2 supports intoxication. Triplicate samples of the cells shown in Fig. 2 were incubated with 10^−10^ M LF_N_-DTA and varying concentrations of PA and cell viability was measured as described in the Fig. 3 legend.

The T118A and D50A mutant receptors were also tested for ability to support intoxication, when expressed in CHO-R1.1 cells, with PA and LF_N_-DTA, a fusion protein with the N-terminal portion of LF fused to the catalytic A chain of diphtheria toxin [10]. For control purposes, these experiments were also performed with the parental CHO-R1.1 cells, and CHO-R1.1 cells expressing a wild-type form (WT) of human ANTXR2-EGFP. These studies revealed that ANTXR2^T118A^ -EGFP supported intoxication albeit less efficiently than the wild-type version of the ANTXR2 protein (Fig. 4A). By contrast, ANTXR2^D50A^-EGFP could not support intoxication (Fig. 4B), an expected result given that this mutation completely disrupts PA binding (Fig. 2).

This report has investigated the roles played by specific metal ion binding residues in the PA-ANTXR2 interaction. As in the case of ANTXR1 [1], the ANTXR2 interaction with wild-type PA is absolutely dependent upon the first receptor MIDAS aspartate (D50). However, unlike ANTXR1, the MIDAS threonine residue (T118) was not essential for PA interaction as judged by PA-binding either to the mutant cell surface receptor (or to the mutant soluble receptor). Moreover the mutant ANTXR2 protein lacking residue T118 was capable of supporting intoxication mediated by wild-type PA.

Taken together, these results demonstrate that there is no absolute requirement for ANTXR2 to adopt an open-like configuration for binding PA. Consistently, unlike ANTXR1 [1], [12] ANTXR2 can bind to, and support intoxication by, mutant PA proteins in which metal-coordinating residue D683 is replaced by either an asparagine or a lysine [13].

The difference in the absolute requirement for an open-like configuration of the receptor MIDAS between ANTXR1 and ANTXR2 is presumably due, at least in part, to the fact that PA binds the ANTXR2 I domain with an approximately 1000-fold tighter binding affinity than it does the ANTXR1 I domain [11], [14]. We propose that it is this higher binding affinity associated with ANTXR2 which overcomes an absolute requirement for an open-like configuration of the receptor I domain.
